# Increase in Gonorrhea Cases in Counties Associated with American Indian Reservations — Montana, January 2012–August 2014

**Published:** 2014-10-17

**Authors:** Randall J. Nett, Peter Choi, Cara Murolo, James S. Murphy

**Affiliations:** 1 Division of State and Local Readiness, CDC; 2 Montana Department of Public Health and Human Services

In May 2012, the Montana Department of Public Health and Human Services noted that 23 cases of gonorrhea were reported in Roosevelt County during October 2011–March 2012, compared with only three cases during January–September 2011. An analysis of surveillance data for Roosevelt County and the six other Montana counties most closely associated with American Indian (AI) reservations showed that, during 2000–2011, the annual incidence rates in the seven counties ranged from 9–43 cases per 100,000, compared with 4–19 cases per 100,000 for all the remaining 49 Montana counties, and 98–129 cases per 100,000 for the United States. Since May 2012, the rates have continued to increase in the seven counties. The 2012 and 2013 incidence rates in counties associated with AI reservations were 74 and 131 cases per 100,000, respectively, compared with four and 10 cases per 100,000 in the remaining counties, and 108 cases per 100,000 in the United States during 2012. This increase in gonorrhea incidence in counties associated with AI reservations began in 2012. During January 2012–August 2014, of the 553 gonorrhea cases reported in Montana, 315 (57%) had a race classification of AI/Alaska Native (AN). In comparison, 6.5% of Montana’s population is classified as AI/AN. Cases were concentrated in few of Montana’s 56 counties; 327 (59%) occurred among residents of seven counties associated with AI reservations that are the home of just 9.8% of Montana’s population. Among all reported Montana cases, the median patient age was 24 years (range = 12–70 years), and 258 (47%) occurred among males. Gonorrhea incidence in Montana counties associated with AI reservations is now comparable to U.S. incidence rates.

Gonorrhea is a sexually transmitted disease (STD) caused by *Neisseria gonorrhoeae* that can cause serious complications in both men and women (1,2).* Sexually active persons at increased risk for gonorrhea are especially those with new or multiple sexual partners, those who use condoms inconsistently or not at all, and those who engage in illicit drug use. Both sexually active females and sexually active males at increased risk for gonorrhea who live in areas of increased transmission should be screened for gonorrhea (1,2). Patients diagnosed with gonorrhea should be treated according to current CDC guidelines (3). Ceftriaxone 250 mg administered intramuscularly in a single dose plus azithromycin (1g) administered orally in a single dose is the preferred treatment regimen for uncomplicated gonorrhea. Sexual contacts of persons with gonorrhea should be identified, examined, tested for the presence of *N. gonorrhoeae* infection, and treated (2).

In response to the increased number of gonorrhea cases, tribal health departments and the Indian Health Service (IHS) have worked to improve STD testing practices in clinical settings; however, further efforts in community outreach and STD testing in nonclinical settings might be required. Challenges to controlling gonorrhea transmission in counties associated with AI reservations include varying coordination of outbreak investigation activities, insufficient staffing and staff turnover, and limited application of STD testing and contact investigation practices.

Steps to reduce gonorrhea transmission in Montana counties associated with AI reservations could include an evaluation and clarification of response roles and training needs among tribal, county, and state health departments, and IHS, and consideration of conducting gonorrhea screening in venues outside of traditional medical clinics (e.g., jails, drug treatment facilities, homes, and schools) (4–7).

## Footnotes

* Additional information available at http://www.cdc.gov/std/gonorrhea.
